# Cancer Communication and User Engagement on Chinese Social Media: Content Analysis and Topic Modeling Study

**DOI:** 10.2196/26310

**Published:** 2021-11-10

**Authors:** Liang Chen, Pianpian Wang, Xin Ma, Xiaohui Wang

**Affiliations:** 1 School of Journalism and Communication Tsinghua University Beijing China; 2 School of Media and Communication Shenzhen University Shenzhen China; 3 Department of Communication University of Maryland Maryland, MD United States; 4 Department of Media and Communication City University of Hong Kong Hong Kong China

**Keywords:** cancer-related information, social media, topic modeling, user engagement, Weibo, cancer

## Abstract

**Background:**

Cancer ranks among the most serious public health challenges worldwide. In China—the world’s most populous country—about one-quarter of the population consists of people with cancer. Social media has become an important platform that the Chinese public uses to express opinions.

**Objective:**

We investigated cancer-related discussions on the Chinese social media platform Weibo (Sina Corporation) to identify cancer topics that generate the highest levels of user engagement.

**Methods:**

We conducted topic modeling and regression analyses to analyze and visualize cancer-related messages on Weibo and to examine the relationships between different cancer topics and user engagement (ie, the number of retweets, comments, and likes).

**Results:**

Our results revealed that cancer communication on Weibo has generally focused on the following six topics: social support, cancer treatment, cancer prevention, women’s cancers, smoking and skin cancer, and other topics. Discussions about social support and cancer treatment attracted the highest number of users and received the greatest numbers of retweets, comments, and likes.

**Conclusions:**

Our investigation of cancer-related communication on Weibo provides valuable insights into public concerns about cancer and can help guide the development of health campaigns in social media.

## Introduction

### Background

Cancer ranks among the most widespread threats to public health worldwide. In China—the world’s most populous country—roughly one-quarter of the population consists of people with cancer. According to the most recent statistics, approximately 4,292,000 individuals have been diagnosed with aggressive cancer, and 2,890,000 people in China died from the disease in 2015 [1].

During the past decade, social media has become an important platform that the public uses to express opinions [2]. The number of Chinese people who use social media specifically to exchange cancer-related information has increased dramatically [3,4]. Among the most popular social media sites in China, Weibo (Sina Corporation)—China’s equivalent of Twitter—has gradually become the primary channel that scholars use to help them understand public concerns about health-related issues. However, due to the massive amounts of data on Weibo and other social media platforms, efficiently extracting public opinions from social media data has emerged as a critical challenge for researchers [5].

In response to this challenge, this study was conducted to investigate how China’s public has discussed cancer on Weibo by summarizing data on the platform via automatic text analytics. First, we conducted topic modeling to categorize cancer-related messages on Weibo. Second, a regression analysis was performed to examine the relationships between various cancer topics and user engagement. The findings could help health care practitioners with designing cancer prevention programs for responding to public concerns, thereby increasing these programs’ effectiveness.

### Literature Review

Traditionally, face-to-face communication between physicians and patients is the primary method of obtaining health information from patients with cancer and their families. However, with the advent of information and communication technologies, an increasing number of people have been using social media to obtain, generate, and share various information about cancer [6]. Social media has become the main channel where individuals can discuss potential causes and treatments of the disease as well as express their thoughts and emotions about cancer (ie, those that they cannot otherwise share with relatives and friends due to social stigmatization) [7]. In addition, a large number of health professionals have considered the availability of health information on the internet to be a positive development and have created and disseminated cancer-related information on social media [8]. Weibo, as one of the most popular social media platforms in China, has also provided a space that Chinese people can use to share and discuss various health-related issues. As such, Weibo is not only a major source of web-based health information; it also provides scholars with unprecedented opportunities to gauge the public’s perceptions and their understanding of health-related issues in China [9,10].

Scholars have conducted content analyses of social media platforms to examine how the public perceives certain diseases, such as H1N1 and breast cancer [11,12]. In recent years, some studies have examined health-related information on social media by using social computing methods in order to explore the general public’s attitudes, thoughts, and feelings toward certain issues in an unobtrusive and comprehensive way [4,13]. For instance, Han et al [14] examined public attitudes and perceptions regarding types of cancer by analyzing cancer-related messages on Twitter via semantic network analysis. They found that breast cancer, lung cancer, and prostate cancer were the three most frequently discussed types of cancer on Twitter. Moreover, discussions about cancer contained positive and negative feelings toward and concerns about cancer.

Although extant research has focused on the number of messages about different types of cancer and the public’s sentiment toward cancer on Twitter, the nature of cancer-related messages on Chinese social media is still understudied. To address this gap in the literature, we used text analytics to answer the following research question: what does the Chinese public talk about when discussing cancer on Weibo (research question 1)?

Some scholars have also investigated the influences that a message’s content has on user engagement when cancer-related messages are disseminated on social media platforms. Social media engagement is measured in terms of users’ interactions with a message. This includes having a conversation with other users through comments and performing actions that demonstrate support, such as liking or retweeting [15,16]. Engagement with a social media message is generally recognized as the most important indicator of popularity and public interest [17]. Some studies have indicated that messages about a specific topic or those that include certain elements generate different levels of user engagement. For example, Wang et al [18] found that messages that describe personal cancer-related experiences resulted in a higher level of engagement (ie, more shares and comments), while cancer prevention–related topics failed to engage social media users. Chen et al [19] also found that social media messages about fear and treatment efficacy attracted more public attention and received a greater number of likes, comments, and retweets. In this study, we investigated the relationships between topics of cancer-related messages and user engagement when such messages are discussed and shared on Weibo. User engagement was measured based on the number of comments, likes, and retweets. Therefore, we proposed the following research question: which cancer topics generate the highest levels of user engagement on Weibo (research question 2)?

## Methods

### Data Collection

We searched for the keyword *cancer* and terms that represented 25 types of cancer to identify cancer-related messages on Weibo that were posted between June 2015 and June 2016. Instead of mining a full year’s worth of data, we randomly selected 7 consecutive weeks and downloaded all tweets that were published during this period by using a Python web crawler. Using consecutive weeks as the sampling frame is one of the most commonly used approaches to obtaining data for content analyses, and this method was confirmed to have relatively good efficiency and accuracy in previous studies [20]. After extracting the data, we formed a data set that contained each message’s content, the time of posting, the number of retweets, the number of comments, the number of likes, and the users who posted each message. We excluded advertisements and messages that were not related to cancer from the data set. The final data set consisted of 16,654 cancer-related messages on Weibo.

Prior to the analysis, we cleaned the data by using the standard preprocessing steps developed in previous studies [21,22]. The procedures included converting the words in messages to lowercase words; removing stop words, punctuation, numbers, and nonword characters; and stemming the remaining text. Afterward, we extracted all nouns from the text by using the *JiebaR* package in R (R Foundation for Statistical Computing), since nouns still carry meaning even without context and are useful for identifying topics in the text of messages on social media. Due to the messages’ language distribution characteristics, we expected a vast number of very infrequent words in the vocabulary of a collection [23]. Hence, we also removed all of the words that appeared fewer than 5 times in the data set. This is one step in the standard procedures for processing textual data [24], and this step can enhance an algorithm’s performance remarkably and stabilize the stochastic inference of latent Dirichlet allocation (LDA) [25].

### Topic Modeling With LDA

Topic modeling algorithms are statistical methods that are used to analyze words in a text to discover the major themes in the text [26]. Topic modeling also enables users to organize and summarize a large number of documents that would be impossible to annotate manually. We used the LDA topic modeling technique, which generated the following two probability distribution outputs: the probability distribution of topics in each document and the probability distribution of terms representing each topic. We determined the number of topics by repeating our analysis with different numbers of topics and comparing the perplexity of each analysis. A lower value of perplexity represents a better model fit [27], and increasing the number of topics generally reduces the perplexity value. When choosing the number of topics, one should consider both the simplicity and interpretability of the textual content [22].

### Regression Analysis

A regression analysis was conducted to investigate how the topics discussed in cancer-related Weibo posts influenced user engagement. The unit of analysis was the text of a Weibo post. The dependent variable was engagement, which was operationalized as the number of retweets, number of comments, and number of likes that a message received. The independent variables were the cancer-related topics that were revealed by the topic modeling analysis. Further, this study considered a series of control variables to control the potential influence that message characteristics had on user engagement, including the number of nouns in a message; whether a message contained a URL, a mention of usernames, or a hashtag; and whether a message was news. The independent variables were entered into the regression model according to their assumed causal order. Specifically, control variables were entered in the first block, and the second block included the topics. The probability distributions generated by the LDA analysis revealed the topic data for each message and the probability of a message falling into a particular topic category. The distribution of the number of nouns was left skewed. Thus, a square root transformation was performed prior to the analysis. The number of retweets, comments, and likes followed a power-law distribution. A log transformation was performed prior to the analysis.

## Results

### Word Frequency Statistics

According to the frequency statistics, the keyword that was mentioned the most often in the data set was *breast cancer* (1465/16,654, 8.80%), followed by *leukemia* (1315/16,654, 7.90%), *esophageal cancer* (1155/16,654, 6.93%), and *lymph cancer* (1121/16,654, 6.73%). Other cancers that were frequently discussed included lung cancer (808/16,654, 4.85%), gastric cancer (761/16,654, 4.57%), cervical cancer (729/16,654, 4.34%), and brain cancer (637/16,654, 3.83%). In summary, discussions about cancer on Weibo focused on the following three cancer categories: digestive cancers (ie, esophageal cancer, gastric cancer, intestinal cancer, gallbladder carcinoma, and liver cancer), urogenital cancers (ie, cervical cancer, metrocarcinoma, endometrial cancer, uterine cancer, bladder cancer, prostate cancer, renal cancer, renal adenocarcinoma, and ovarian cancer), and lymphoid and hematopoietic cancers (ie, leukemia, lymph cancer, and lymphoma). In addition, a large part of the discussions referred to cancer and malignancy in a more general sense.

### Topic Modeling via LDA

By conducting topic modeling to analyze the text and comparing the perplexity indices, we found that categorizing topics into 6 topic categories was optimal. Based on our findings, as well as findings from previous cancer-related studies [27], we identified the following six topics.

#### Topic 1: Social Support

The topic of social support encompassed a wide range of opinions about forms of social support and available help for coping with cancer. The most commonly used keywords that represented social support were *child*, *hope*, *love*, *friend*, *father*, *mother*, *classmate*, *son*, *daughter*, and *family*. In addition, the keyword *leukemia* was also found in discussions about social support, largely because relatives and friends of people with leukemia tend to seek social support on Weibo.

#### Topic 2: Cancer Treatment

The topic of cancer treatment represented discussions involving the keywords *treatment*, *tumor*, *early stage*, *later stage*, *symptom*, *patient*, *surgery*, *hospital*, *method*, *chemotherapy*, *diagnose*, and *appearance* and other words that immediately suggested the topic of cancer treatment.

#### Topic 3: Cancer Prevention

The topic of cancer prevention generally concerned issues related to cancer prevention, especially food and eating habits. The most frequently used keywords that represented this topic were *risk*, *food*, *foodstuff*, *research*, *vegetables*, *diet*, *prevention*, *fruit*, *anticancer*, *drinking*, *chance*, *intake*, *soy milk*, *cured food*, *vitamins*, and *fat*. As such, this topic covered not only cancer prevention but also relationships among food, eating habits, and cancer risks.

#### Topic 4: Women’s Cancers

The most frequently used keywords that represented the specific characteristics of women’s cancers were *female*, *uterus*, *breast cancer*, *cervical cancer*, *HPV*, *age*, *menstruation*, *fertility*, and *ovarian cancer*. Discussions about this topic included several common types of women’s cancers and important related topics, such as the human papilloma virus, menstruation, fertility, and age.

#### Topic 5: Smoking and Skin Cancer

This topic represented smoking-related cancers and skin cancer in particular. The most frequently used keywords included *secondhand smoke*, *skin cancer*, *lung cancer*, *smoker*, *sunscreen*, *risk*, *quit smoking*, *ultraviolet light*, and *child*.

#### Topic 6: Others

We classified the sixth topic as “others,” since it contained several topics related to cancer prevention, men’s cancers, alcohol, and other types of cancer. The most frequently used keywords were *golden rules for cancer prevention*, *exercise*, *cell phone*, *male*, *prostate*, *BMI*, *heart*, *beer*, *drinking*, *oral cancer*, and *intestinal*.

**Figure 1:**
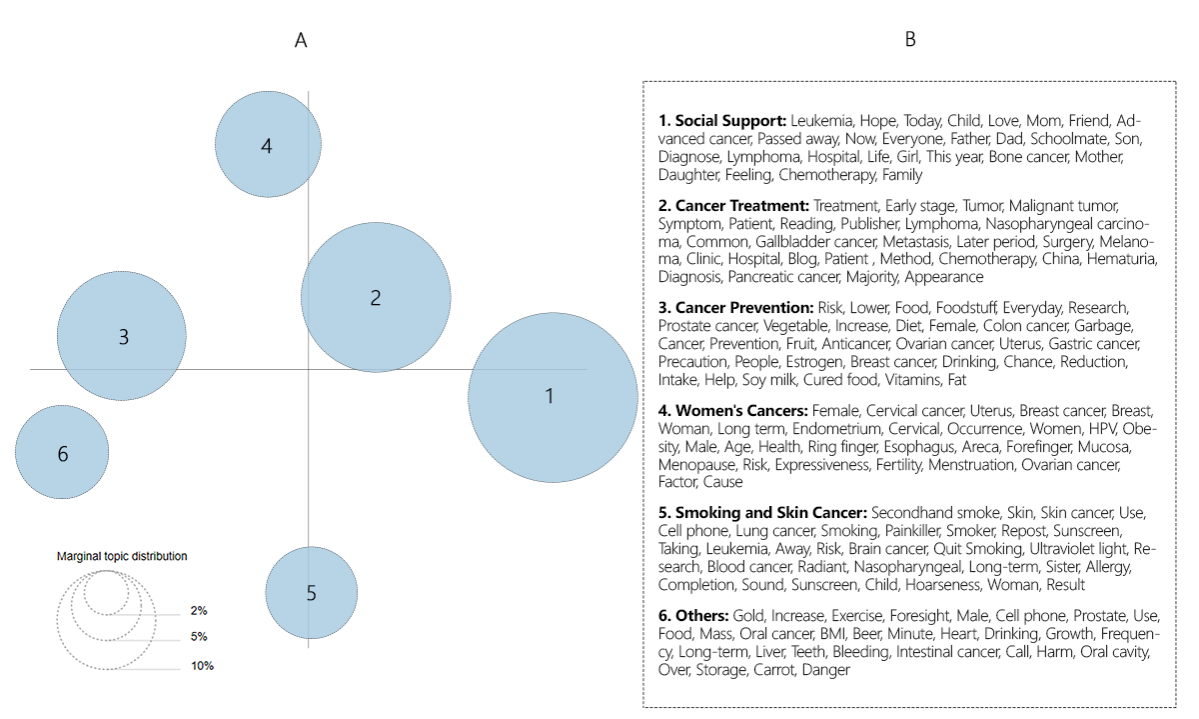
Intertopic distance map. A: LDAvis package and the 30 most common words representing each topic; B: translated from Chinese terms. The size of the circles indicates the topic proportions for all text. HPV: Human Papilloma Virus.

Figure 1 shows the intertopic distance of all topics.

### Descriptive Analysis of Engagement

Table 1 presents the descriptive statistics and intercorrelations of the three measures of engagement. The number of retweets for all of the messages varied from 0 to 8033 (mean 10.73, SD 169.54). The number of comments ranged from 0 to 10,897 (mean 6.38, SD 142.08). The number of likes ranged from 0 to 48,963 (mean 20.21, SD 516.83). The distribution of all of the engagement measures followed a long-tail distribution; a few messages generated a high level of engagement, while most of the others languished in obscurity. Moreover, the correlation tests showed a strong correlation between the number of retweets and comments, while the binary relationships between these two popularity indices and the number of likes were moderate. This indicated that the three measures were highly related and presented unique aspects of user engagement.

**Table 1:** Descriptive analysis and correlation matrix of user engagement measures.

Popularity measures			Number of retweets^a^	Number of comments^b^		Number of likes^c^	
**Number of retweets**							
		*r*	1		0.732^d^		0.414^d^
		*P* value	—^e^		<.001		<.001
**Number of comments**							
	*r*		0.732^d^	1		0.484^d^	
	*P* value		<.001	—		<.001	
**Number of likes**							
	*r*		0.414^d^	0.484^d^		1	
	*P* value		<.001	<.001		—	

^a^The mean number of retweets was 10.73 (SD 169.54).

^b^The mean number of comments was 6.38 (SD 142.08).

^c^The mean number of likes was 20.21 (SD 516.83).

^d^Significant at the *P*<.001 level.

^e^Not applicable.

### Regression Analysis Results

Table 2 presents the results from the ordinary least squares regression analysis for predicting engagement. All factors were significantly associated with the number of retweets, and social support was the primary factor related to the number of retweets. In addition, social support, cancer treatment, women’s cancers, and smoking and skin cancer were positively related to the number of comments. The topic of cancer prevention was not significantly associated with the number of comments on messages (*P*=.09). It means that the coefficient of it (.016) is not significant. Besides, all factors were positively associated with the number of likes, except for the inclusion of URLs (*P*=.07) and the cancer prevention topic (*P*=.06).

The results of the regression analysis indicated that among all five cancer topics, discussions about social support had the greatest effect on user engagement indices, that is, the number of retweets (*β*=.26; *P*<.001), comments (*β*=.36; *P*<.001), and likes (*β*=.34; *P*<.001). In other words, social support was the most popular cancer topic on Weibo. Messages that contained discussions about social support were more likely to attract users’ attention and stimulate engagement on social media. Further, the cancer treatment and smoking and skin cancer topics were also strong predictors of user engagement, which suggests that these topics attract a lot of public attention.

**Table 2:** Regression results based on user engagement measures as the criteria (N=16,651).

Variables		Number of retweets^a^, *β*^b^	Number of comments^c^, *β*^b^	Number of likes^d^, *β*^b^
Intercept		.001	.001	0
**Control variables**				
	Noun count	.118^e^	–.005^e^	.056^e^
	Inclusion of a URL	.066^e^	–.050^e^	.014^e^
	Inclusion of a username mention	.044^e^	.028^e^	.023^f^
	Inclusion of a hashtag	.114^e^	.057^e^	.126^e^
	The message was news	.144^e^	.042^e^	.108^e^
**Topics**				
	Social support	.262^e^	.361^e^	.337^e^
	Cancer treatment	.125^e^	.097^e^	.116^e^
	Cancer prevention	.033^e^	.016^e^	.023^e^
	Women’s cancers	.054^e^	.038^e^	.059^e^
	Smoking and skin cancer	.110^e^	.084^e^	.123^e^

^a^The *R*^2^ value for the number of retweets is 0.066 (*P*<.001).

^b^Indicates the *β* weights or standardized regression weights.

^c^The *R*^2^ value for the number of comments is 0.089 (*P*<.001).

^d^The *R*^2^ value for the number of likes is 0.075 (*P*<.001).

^e^Significant at the *P*<.001 level.

^f^*P*=.002

## Discussion

### Principal Findings

Our study made an initial attempt at conducting a systematic textual analysis of cancer-related messages on Weibo. By using word frequency statistics and conducting topic modeling and regression analyses, we identified the major topics of public discussions about cancer on Chinese social media and examined the relationship between different cancer topics and user engagement.

The word frequency statistics indicated that the cancer that is discussed the most often on Weibo is breast cancer, followed by leukemia, esophageal cancer, and lymphoma. This contradicts the incidence and mortality rates of cancer in China [1], where lung cancer, stomach cancer, and liver cancer are the most prevalent cancers, followed by breast cancer. A plausible explanation for this is that the survival rate of patients with breast cancer is greater than the survival rates of most patients with other types of cancer. Consequently, many patients with breast cancer and their families might spend a longer time expressing their opinions and seeking relevant information on Weibo [28,29]. In addition, according to Weibo’s annual report, 68.8% of Weibo users are aged 18-35 years, 77.8% of users have a high level of education, and users in high-income areas account for a relatively large proportion of users [30]. In other words, a majority of Weibo users are young people with high socioeconomic statuses. However, those with a higher risk of cancer and mortality are typically older adults and people with low socioeconomic statuses. As such, we found a difference between the cancers discussed on Weibo and the cancers with the highest mortality rates in China.

We used automatic text analytics to examine cancer-related discussions on Weibo. The discussions were categorized into the following six topics: social support, cancer treatment, cancer prevention, women’s cancers, smoking and skin cancer, and other topics. Our topic categories summarize the main topics that are discussed by the public when they talk about cancer, and they are statistically supported by a large amount of data. In particular, the results revealed that people mainly seek and provide social support messages about cancer on Weibo. Moreover, our regression results confirm that social support is the most popular cancer topic on Weibo. Messages about social support received a larger number of retweets, comments, and likes than those about other topics. These results align with findings from previous studies [29,31] and can possibly be explained by the Chinese collectivist culture, in which people’s decision-making is usually interdependent. When developing medical responses to health problems, Chinese people tend to rely on advice and support from health care professionals and people with similar health conditions [32].

Our results also suggest that many public discussions about cancer on Chinese social media focus on cancer treatment. In addition, messages about cancer treatment received a large number of retweets, comments, and likes. This suggests that most Weibo users pay more attention to (ie, retweet, comment on, and like) messages that discuss cancer symptoms and treatments and pay less attention to other topics, such as cancer prevention. However, a closer look at the discussions indicates that most messages on Weibo exaggerate the effects that diet has on cancer treatment. For instance, the following message captures this tendency quite well:

[Onion wine is effective in treating cancer] Tu Ge is a 40-year-old man. After diagnosed with cancer, he drank onion wine every day. Then, the cancer cells are getting smaller.

Although such messages on Weibo attract a lot of attention and generate a lot of discussion, such inaccurate statements can misguide individuals’ responses to cancer [33]. Further, while health-related misinformation is a long-standing area of research [34], studies on the effects of nonmalicious misinformation remain scarce. Future studies could investigate specific categories of misinformation about cancer, especially the difference between malicious and nonmalicious cancer-related misinformation.

Interestingly, our results show that while people do not discuss smoking and skin cancer very often on Weibo, messages about smoking-related topics are more likely to generate a high level of user engagement. One possible explanation for this could be that unlike other cancer topics, such as cancer treatment and prevention, that attract people living with cancer, smoking is a common issue in China. As a result, smokers and their relatives and friends may be interested in sharing and engaging in smoking-related discussions. Thus, smoking-related messages generate a higher level of user engagement on Weibo.

Our findings provide several insights. First, the thematic framework that we discovered via topic modeling could complement and verify the traditional categorization of cancer-related topics. The categorization of public discussions also sheds light on the public’s knowledge and awareness of cancer-related issues [35]. Second, by combining automatic text analytics and regression analyses, we provided an approach to summarizing and gaining insights from a large amount of user-generated content. In terms of practicality, this means that the topic modeling of cancer-related content on Weibo can be conducted to monitor the public’s attention to cancer in real time. Third, the regression analysis showed that the social support and cancer treatment topics attracted the most public attention. These findings could help relevant agencies and health care professionals to understand social media users’ needs and suggest strategies for designing effective health campaigns and promotions.

### Limitations and Suggestions for Future Research

Our study has several limitations. First, because data on social media change greatly over time, the messages posted at the time of this study may not fully reflect the current situation. Additionally, since our analysis did not account for the time of posting, the dynamic nature of the public’s attention to cancer-related topics remains unknown. Especially since the State Council of China issued the guideline on Internet Plus Healthcare in 2018, the health information industry in China has witnessed rapid growth and structural changes. Therefore, on the basis of this study, we call for further studies on this topic to compare discussions conducted at different time points to gain insights into how health-related discussions on social media will evolve over time.

Second, it is possible that the cancer-related posts of users who are more popular on social media platforms attracted more attention. Thus, the number of followers that an individual Weibo user has could be a strong confounder of the number of retweets, comments, and likes. However, this factor was not taken into consideration in this study due to the lack of techniques and resources. Future studies can conduct hierarchical regression analyses to investigate this issue.

Finally, we analyzed data from Weibo only. Therefore, any generalization of our results should take the characteristics of Weibo users into consideration [36]. Future research should be conducted to analyze data on other social media platforms and cross-verify our results.

## Abbreviations

LDA: latent Dirichlet allocation
